# Supplementary figures and images for: EZH2 Inhibition Sensitizes IDH1R132H-Mutant Gliomas to Histone Deacetylase Inhibitor
Source: Cells. 2024 Jan 25;13(3):219. doi: 10.3390/cells13030219 (PMC10854521; doi:10.3390/cells13030219)

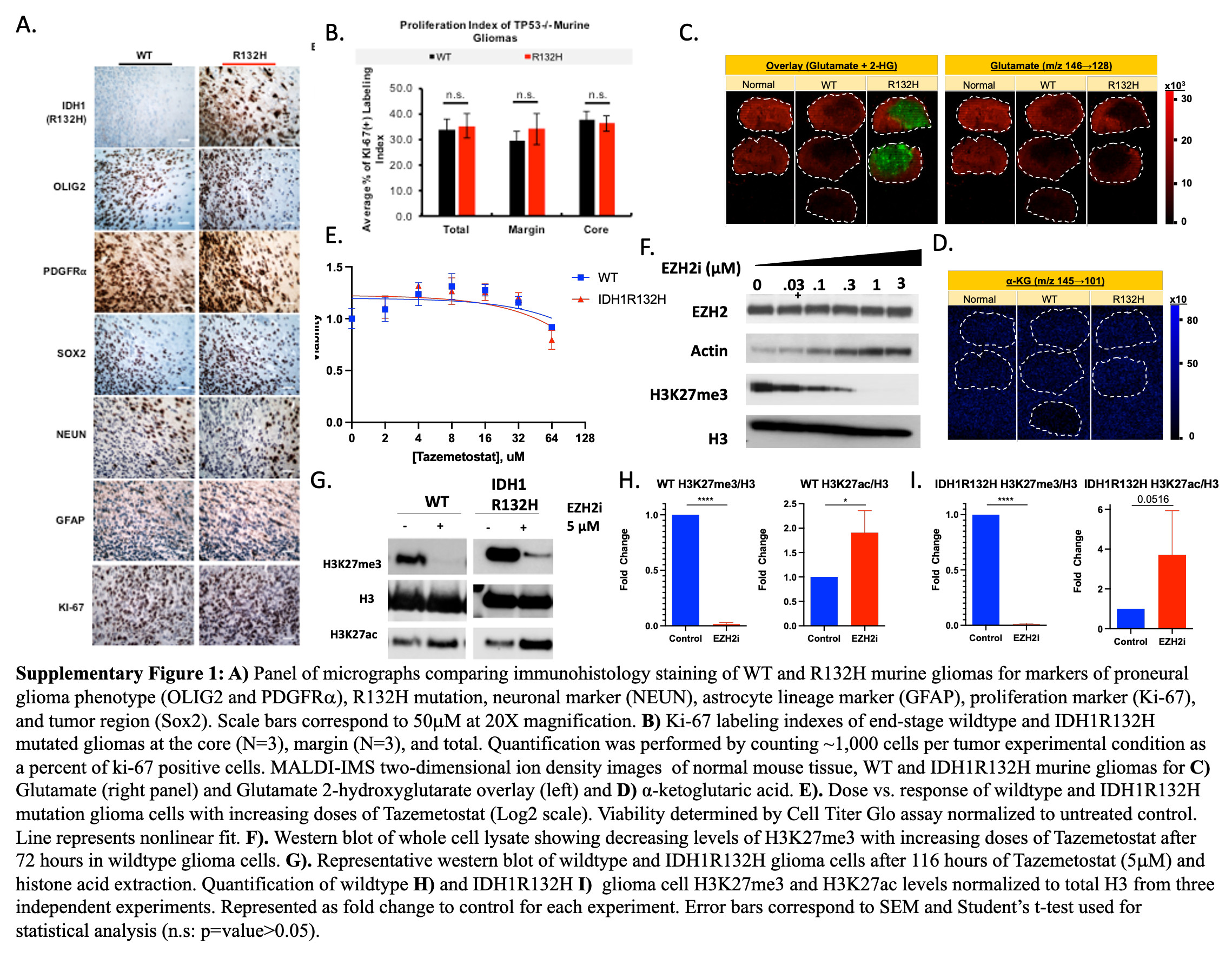

Supplement: Supplementary file 1 [file cells-13-00219-s001.zip › SupplementaryFig1.jpg]

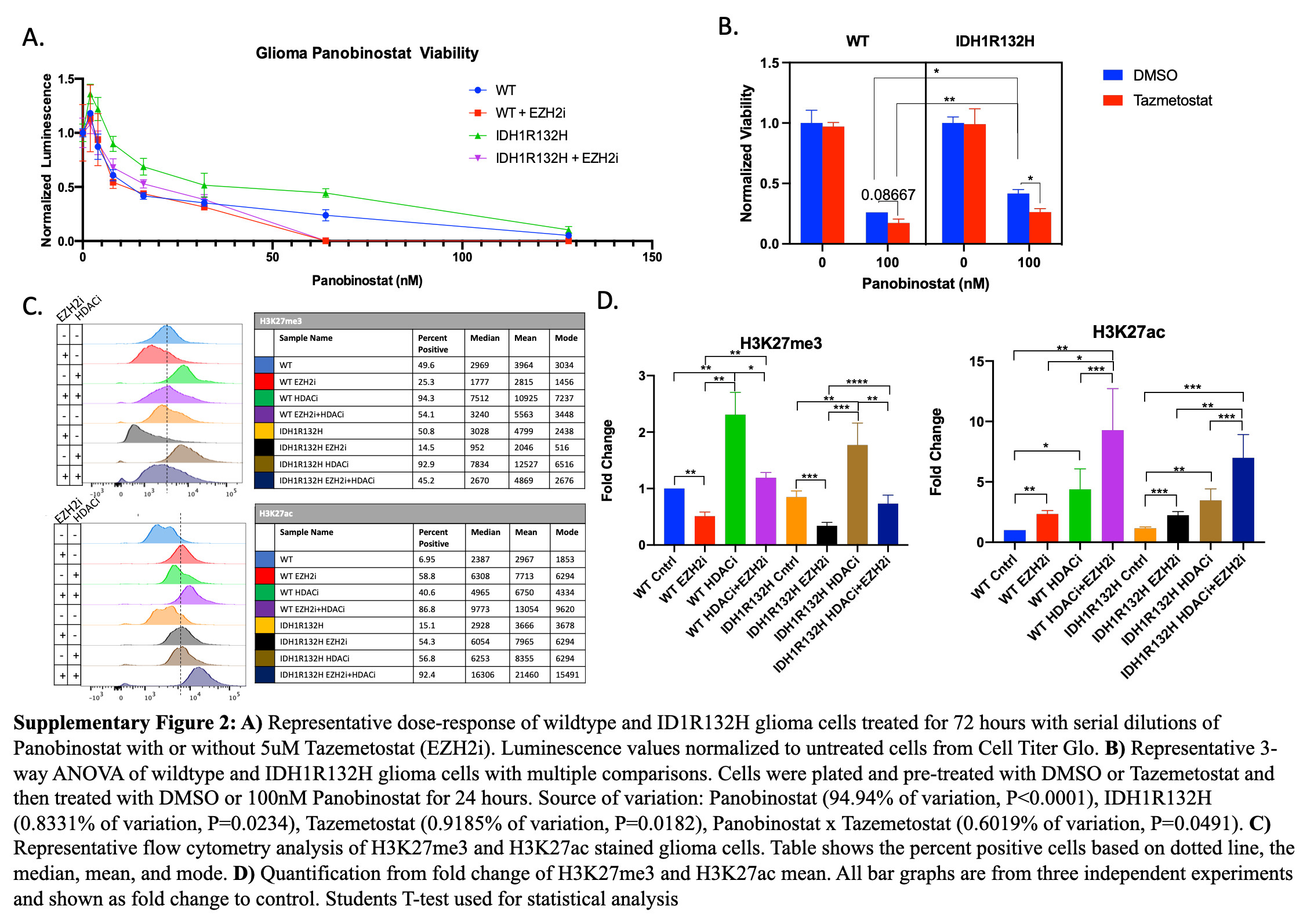

Supplement: Supplementary file 1 [file cells-13-00219-s001.zip › SupplementaryFig2.jpg]

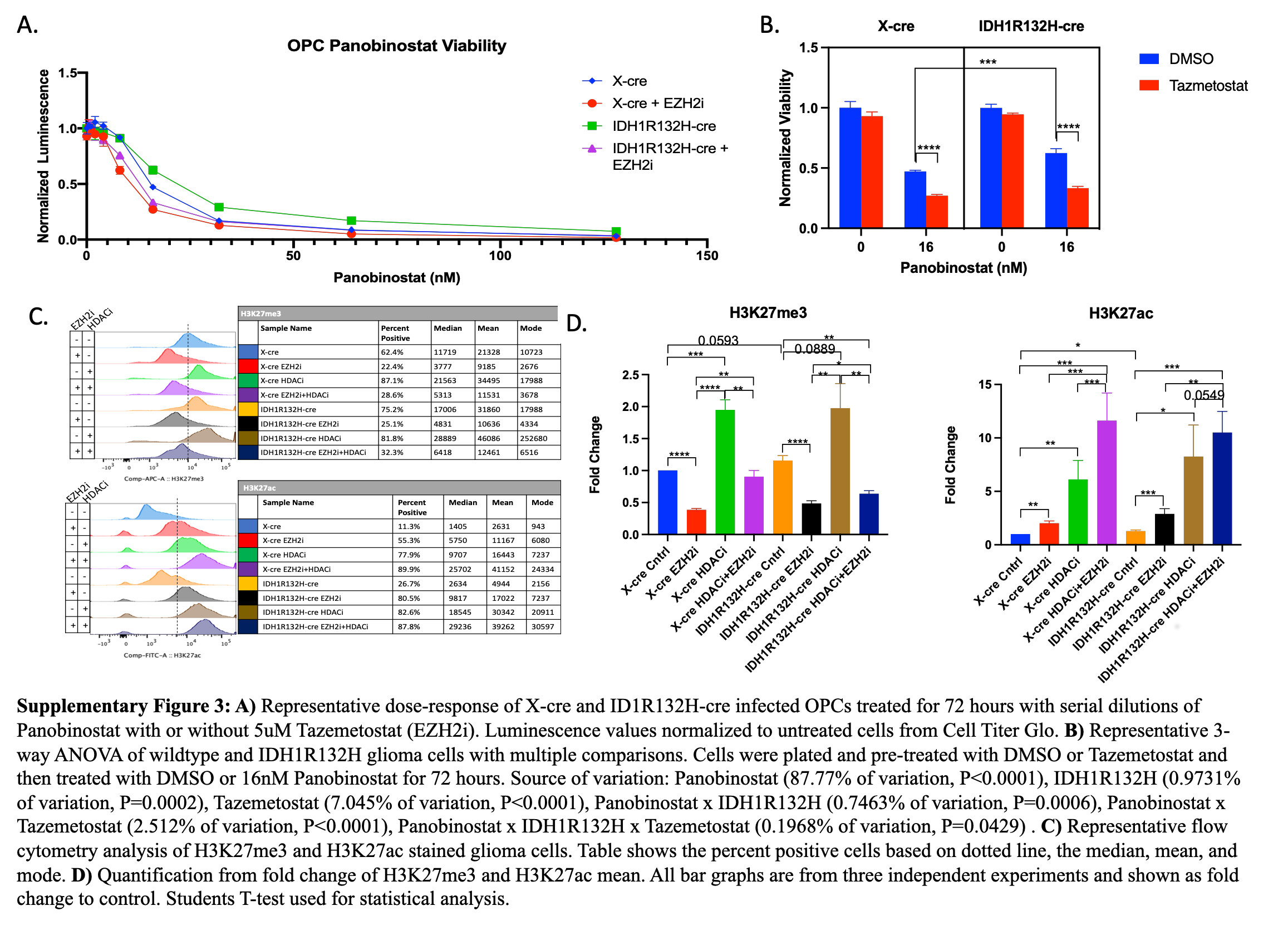

Supplement: Supplementary file 1 [file cells-13-00219-s001.zip › SupplementaryFig3.jpg]
